# Supplementary material for: Sugar transporters enable a leaf beetle to accumulate plant defense compounds
Source: Nat Commun. 2021 May 11;12:2658. doi: 10.1038/s41467-021-22982-8 (PMC8113468; doi:10.1038/s41467-021-22982-8)
Supplement: Supplementary file 1 — Supplementary Information [file 41467_2021_22982_MOESM1_ESM.pdf]

Supplementary Information

Sugar transporters enable a leaf beetle to accumulate plant defense compounds

Zhi-Ling Yang[1], Hussam Hassan Nour-Eldin[2], Sabine Hänniger[3], Michael Reichelt[4], Christoph Crocoll[2], Fabian Seitz[1], Heiko Vogel[3] & Franziska Beran[1]

[1]Research Group Sequestration and Detoxification in Insects, Max Planck Institute for Chemical Ecology, Jena, Germany.

[2]DynaMo Center, Department of Plant and Environmental Sciences, Faculty of Science, University of Copenhagen, Frederiksberg, Denmark.

[3]Department of Entomology, Max Planck Institute for Chemical Ecology, Jena, Germany.

[4]Department of Biochemistry, Max Planck Institute for Chemical Ecology, Jena, Germany

This pdf file includes:

Supplementary Figures 1-10

Supplementary Tables 1-5

Supplementary Figures

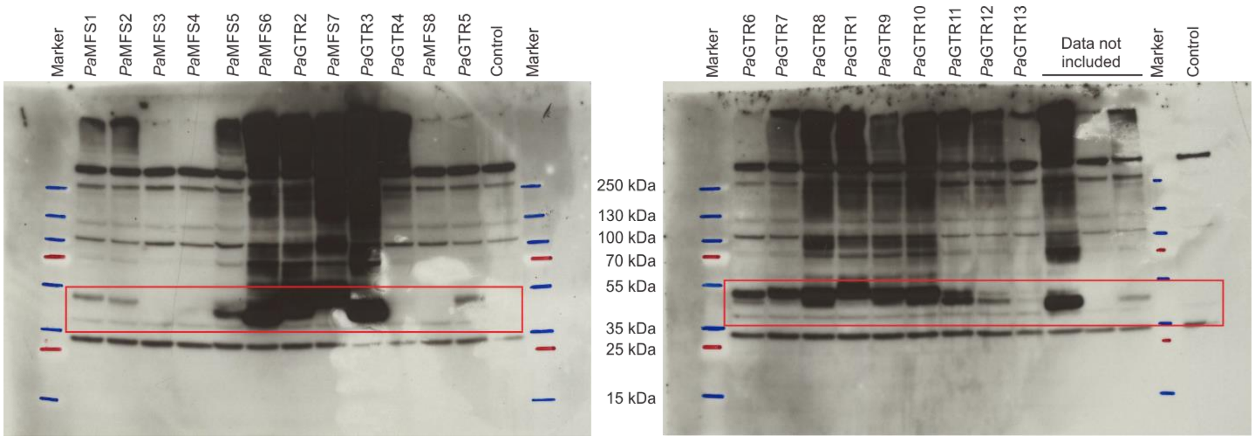

Supplementary Figure 1. Detection of recombinant transporters expressed in High Five insect cells by Western blotting. The red rectangles highlight blot regions with recombinant proteins. Protein marker bands are marked with blue and red lines on the film. Heterologous expression of candidate transporters in insect cells followed by Western blotting was repeated three times.

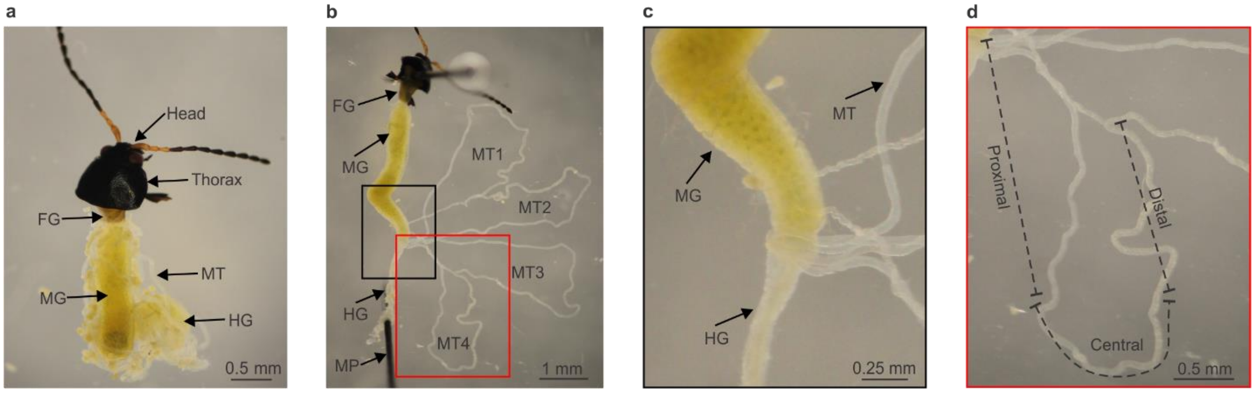

Supplementary Figure 2. Morphology of the Malpighian tubule system of *P. armoraciae*. a Gut with Malpighian tubules dissected from a four-day old adult *P. armoraciae* beetle. **b** Malpighian tubule system consisting of four tubules (MT1-4) that empty at their proximal ends near the midgut-hindgut junction. Two tubules each fuse at their distal ends and appear to be attached to the midgut. **c** Magnification of the midgut-hindgut junction framed in black in panel **b**. **d** Magnification of one tubule (MT4) framed in red in panel **b**. Expression of candidate transporters was analyzed in the proximal, central and distal sections (marked with dotted lines) by quantitative PCR (shown in Fig. 1e). FG, foregut; MG, midgut; HG, hindgut; MT, Malpighian tubules; MP, metal pin. Three different beetles were dissected to establish the morphology of the Malpighian tubule system.

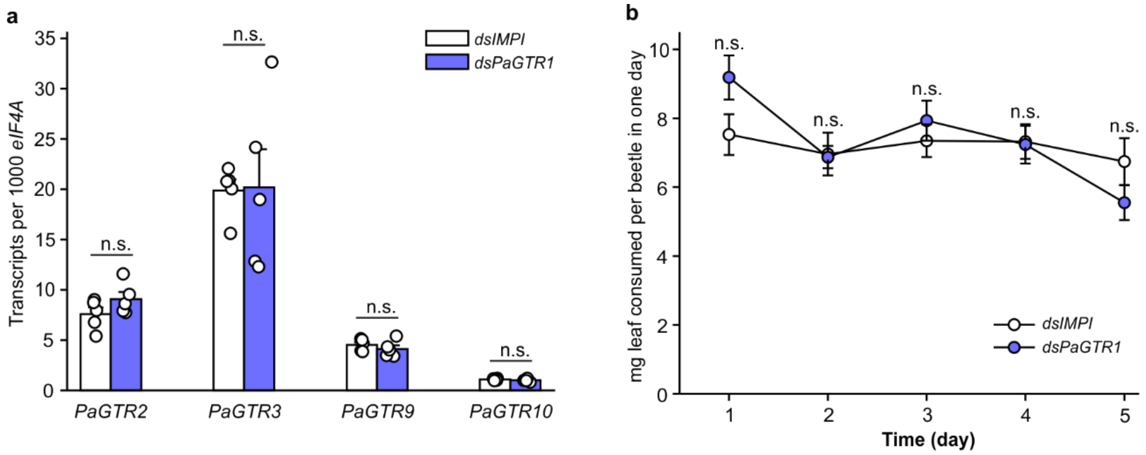

Supplementary Figure 3. Analysis of potential off-target effects of *dsPaGTR1*-injection and comparison of beetle feeding on *Arabidopsis* leaves. a Four days after dsRNA injection, the gene expression of *PaGTR2*, *PaGTR3*, *PaGTR9* and *PaGTR10* was determined by quantitative PCR to assess whether there was off-target silencing on other *PaGTR*s. The nucleotide sequence of *PaGTR1* was most similar to *PaGTR9* and *PaGTR10* in the *P. armoraciae* transcriptome. Recombinant *Pa*GTR2, *Pa*GTR3 and *Pa*GTR9 were also active towards indol-3-ylmethyl glucosinolate (n = 5 biological replicates, three beetles per replicate). **b** Four days after dsRNA injection, adults were allowed to feed on *Arabidopsis* leaves for five days (n = 10 biological replicates, feeding damage of five beetles per replicate). Treatments were compared by two-tailed Student's t-test or Mann-Whitney U test. Data are shown as mean ± s.e.m. n.s., not significantly different. P values are included in Supplementary Data 6.

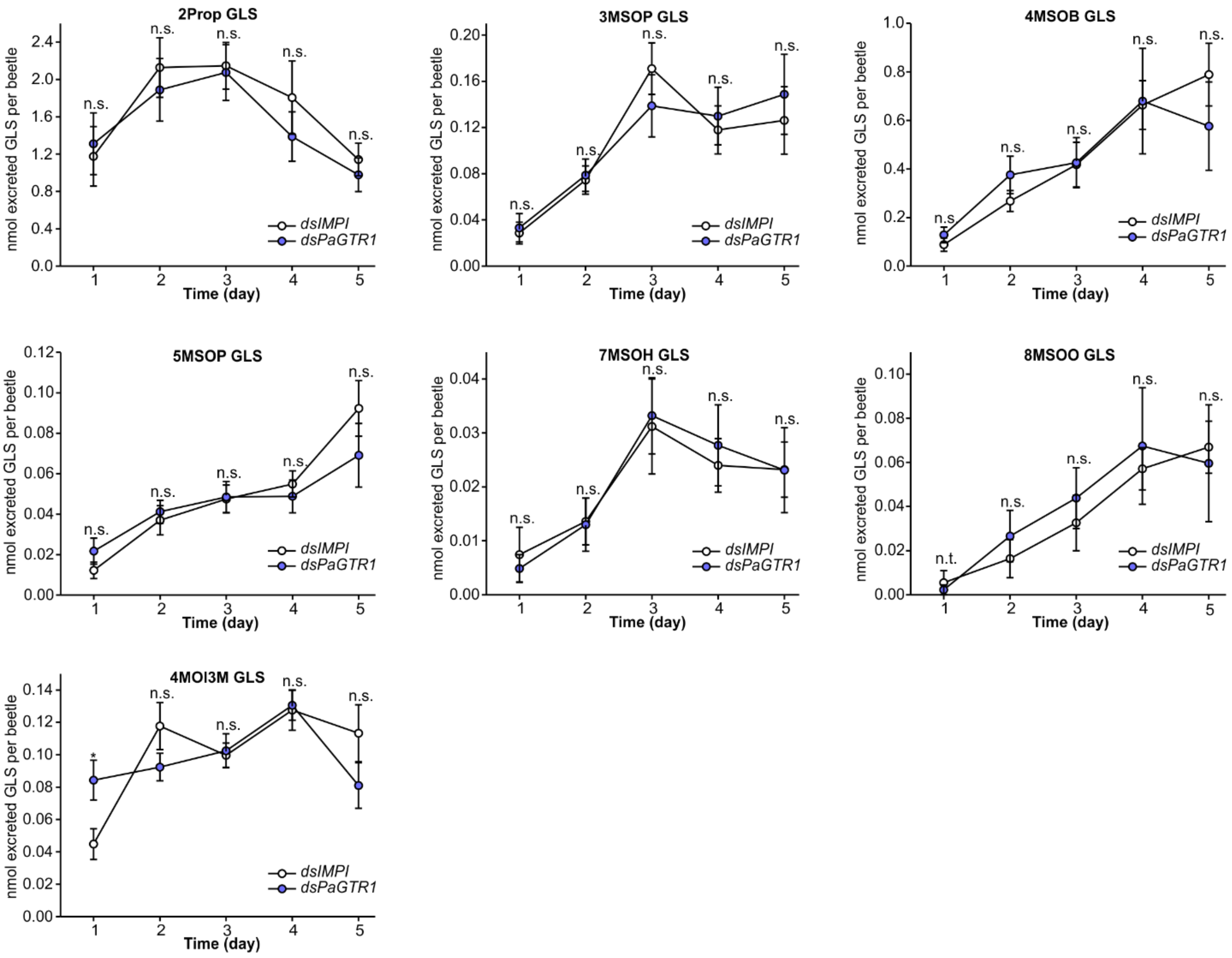

Supplementary Figure 4. Time course of glucosinolate excretion during feeding on *Arabidopsis* by adult *P. armoraciae* after *dsIMPI*- or *dsPaGTR1*-injection. The excreted glucosinolate amounts on each day were compared by two-tailed Student's t-test or Mann-Whitney U test (n = 9-10 biological replicates, feces of five beetles per replicate). Data are shown as mean ± s.e.m. n.t., not tested; n.s., not significantly different; * $P < 0.05$; 2Prop, 2-propenyl; 3MSOP, 3-methylsulfinylpropyl; 4MSOB, 4-methylsulfinylbutyl; 5MSOP, 5-methylsulfinylpentyl; 7MSOH, 7-methylsulfinylheptyl; 8MSOO, 8-methylsulfinyloctyl; 4MOI3M, 4-methoxyindol-3-ylmethyl. P values are included in Supplementary Data 6.

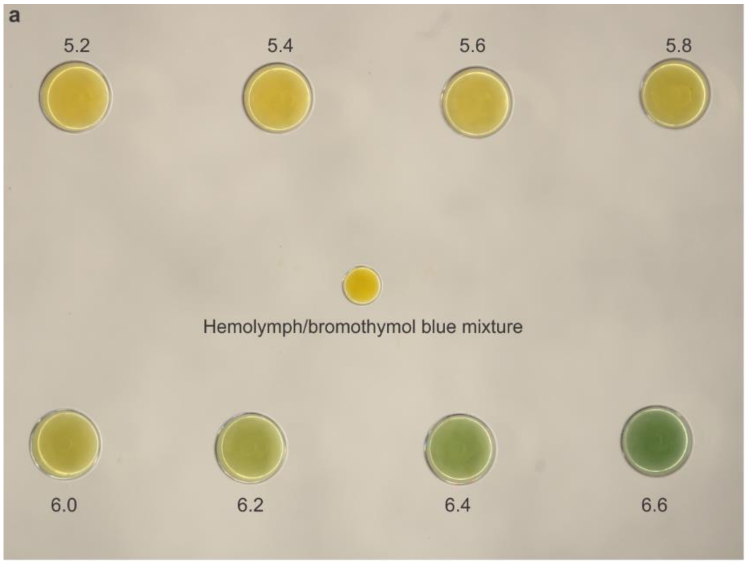

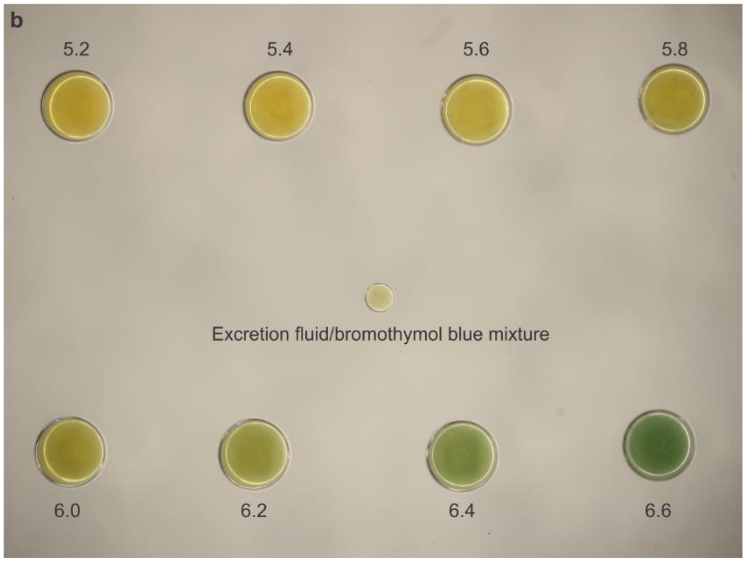

Supplementary Figure 5. pH of hemolymph (a) and excretion fluid of isolated Malpighian tubules (b) of *P. armoraciae* adults. Hemolymph and excretion fluid were mixed with an equal volume of 0.16% (w/v) bromothymol blue. Buffered standard solutions from pH 5.2 to 6.6 containing 0.08% bromothymol blue are shown as reference. The analysis was performed with three independent collections of hemolymph and excretion fluid, respectively.

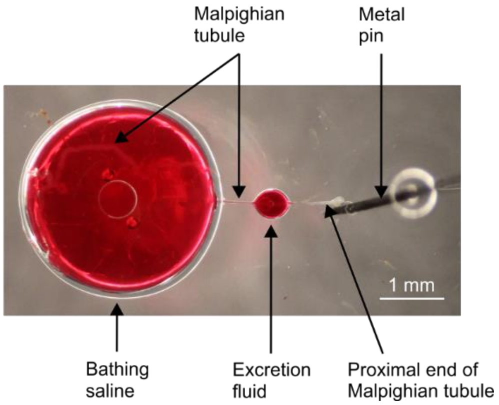

Supplementary Figure 6. Preparation of *P. armoraciae* Malpighian tubule for glucoside excretion assay (Ramsay assay). The dissected Malpighian tubule was placed in a droplet of bathing saline under water-saturated paraffin-oil, the proximal end was drawn out of the droplet, attached to the Sylgard-coated petri dish with a metal pin, and cut to allow the collection of excretion fluid. A mixture of eight different plant glucosides, each at a concentration of 6.7 mM, and 0.1% (w/v) amaranth was added to the saline. After 2-3 h, the bathing saline, Malpighian tubule and excretion fluid were sampled and the glucoside composition analyzed by LC-MS/MS. A total of 36 Ramsay assays were performed of which 11 could be analyzed because a sufficient amount of excretion fluid was formed.

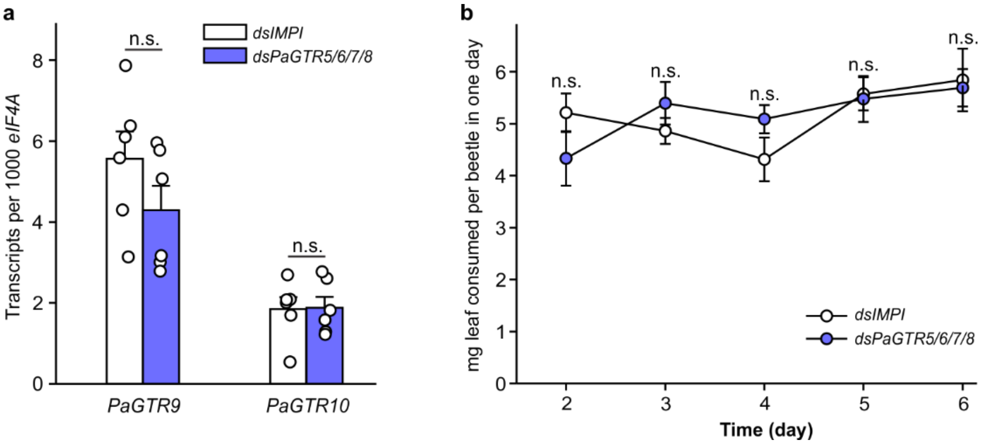

Supplementary Figure 7. Analysis of potential off-target effects of *dsPaGTR5/6/7/8*-injection and comparison of beetle feeding on *Arabidopsis* leaves. a Six days after dsRNA injection, the gene expression of *PaGTR9* and *PaGTR10* was determined by quantitative PCR to assess whether there was off-target silencing on other *PaGTR*s. The nucleotide sequence of *PaGTR5/6/7/8* was most similar to *PaGTR9* and *PaGTR10* in the *P. armoraciae* transcriptome (*n* = 6 biological replicates, two beetles per replicate). **b** After dsRNA injection, adults were allowed to feed on *Arabidopsis* leaves for six days. Fed amounts of each day were recorded from the second to the sixth day (*n* = 10 biological replicates, feeding damage of six beetles per replicate). Treatments were compared by two-tailed Student's *t*-test. Data are shown as mean ± s.e.m. n.s., not significantly different. *P* values are included in Supplementary Data 6.

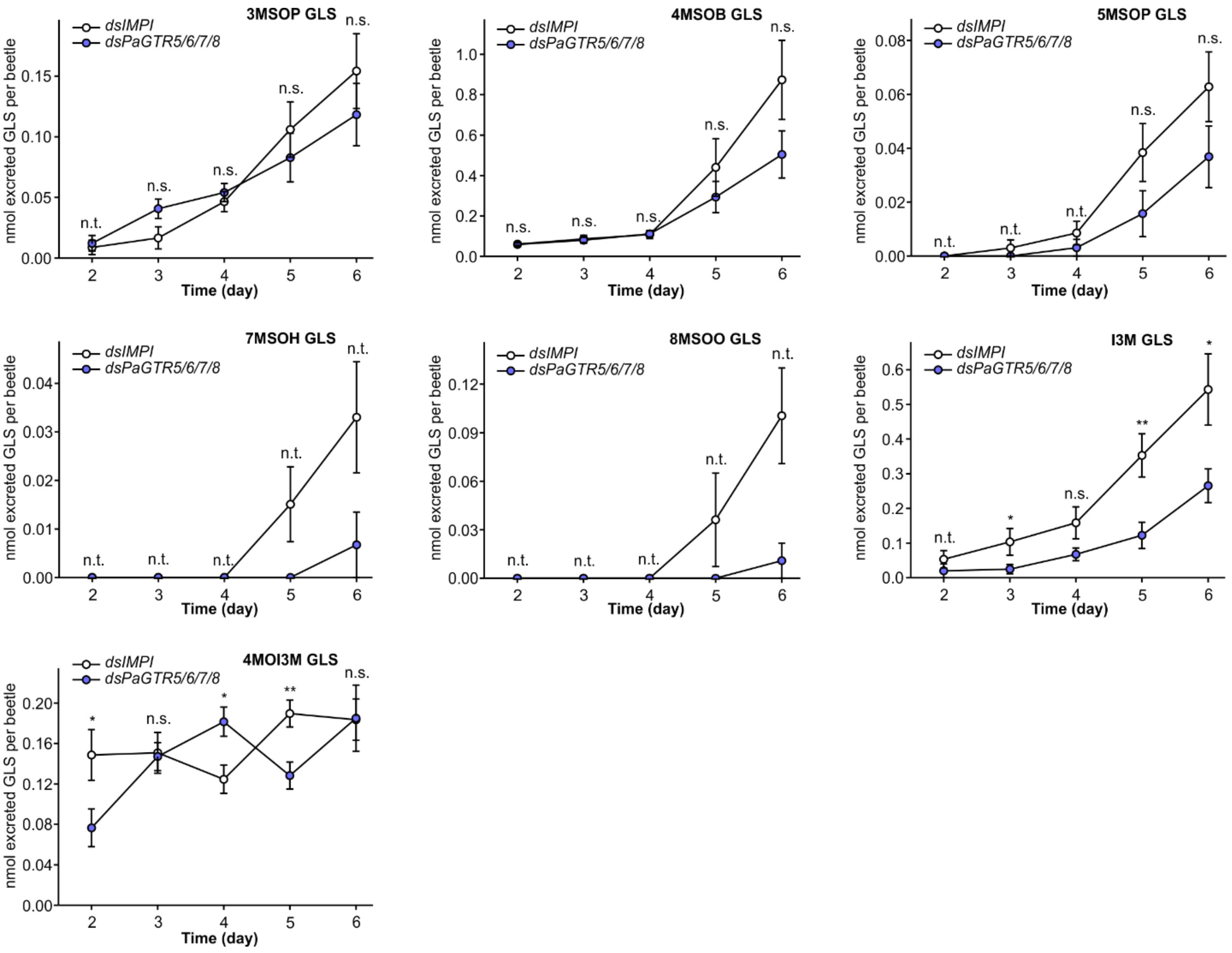

Supplementary Figure 8. Time course of glucosinolate excretion during feeding on *Arabidopsis* by adult *P. armoraciae* after *dsIMPI*- or *dsPaGTR5/6/7/8*-injection. Excreted glucosinolate amounts per day were compared by two-tailed Student's *t*-test or Mann-Whitney *U* test (n = 10 biological replicates, feces of six beetles per replicate). Data are shown as mean ± s.e.m. n.t., not tested; n.s., not significantly different; *P < 0.05; **P < 0.01; 3MSOP, 3-methylsulfinylpropyl; 4MSOB, 4-methylsulfinylbutyl; 5MSOP, 5-methylsulfinylpentyl; 7MSOH, 7-methylsulfinylheptyl; 8MSOO, 8-methylsulfinyloctyl; I3M, indol-3-ylmethyl; 4MOI3M, 4-methoxyindol-3-ylmethyl. *P* values are included in Supplementary Data 6.

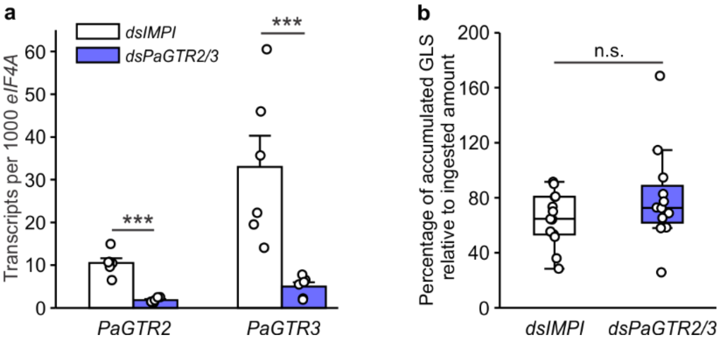

Supplementary Figure 9. Effect of *dsPaGTR2/3* injection on gene expression and accumulation of ingested glucosinolates in adult *P. armoraciae* beetles. Expression of *PaGTR2* and *PaGTR3* was analyzed in *dsIMPI*- and *dsPaGTR2/3*-injected beetles by quantitative PCR (n = 6 biological replicates, two beetles per replicate). Data are shown as mean ± s.e.m. **b** Accumulation of ingested glucosinolates in adult *P. armoraciae* beetles. Adults were fed for one day with *Arabidopsis* leaves, starved for one day, and collected for glucosinolate analysis. Accumulated glucosinolates (GLS): sum of 3-methylsulfinylpropyl GLS, 3-methylthiopropyl GLS, 4-methylsulfinylbutyl GLS, 4-methylthiobutyl GLS, 7-methylsulfinylheptyl GLS and 8-methylsulfinyloctyl GLS. Due to a high background of indolic GLS in beetles, it was not possible to quantify the accumulation of ingested indolic GLS from *Arabidopsis*. Box plots show the median, interquartile range, and outliers of each data set (n = 12-13 biological replicates, three beetles per replicate). Treatments were compared by two-tailed Student's t-test. n.s., not significantly different; ***$P < 0.001$. P values are included in Supplementary Data 6.

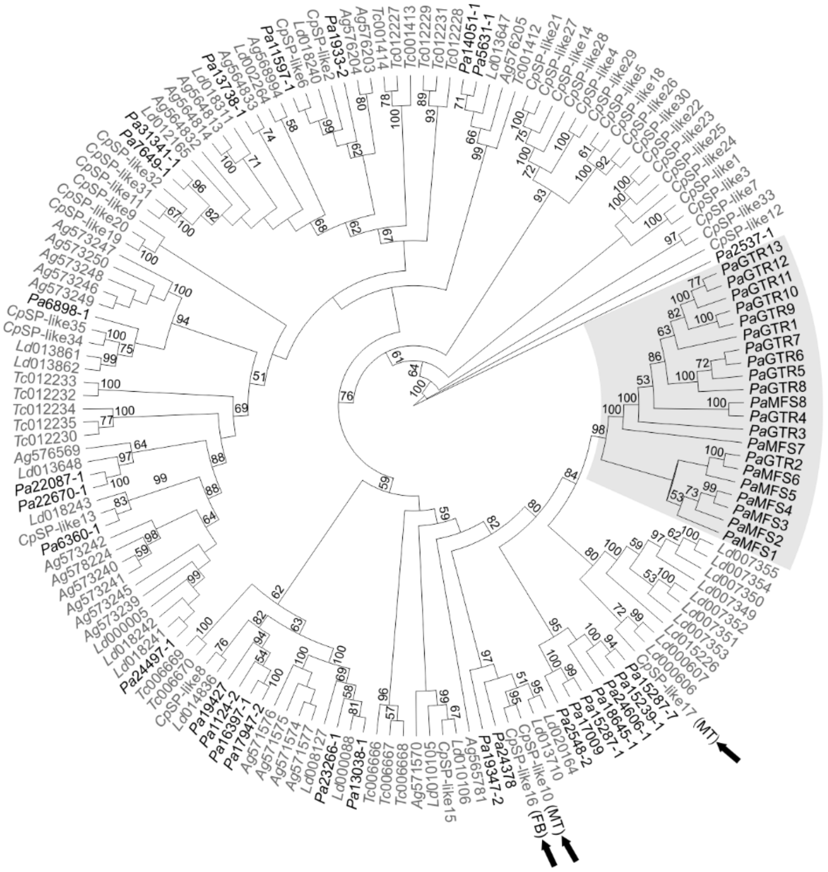

Supplementary Figure 10. Diversification of coleopteran sugar porters. Maximum-likelihood inferred phylogeny of a subset of predicted coleopteran sugar porters (Transporter Classification Database ID 2.A.1.1) identified in the *P. armoraciae* transcriptome (written in black) and the genomes of *Leptinotarsa decemlineata*, *Anoplophora glabripennis*, and *Tribolium castaneum* (marked with a black frame in Supplementary Data 3), and 35 sugar porters identified in the proteome of *Chrysomela populi* (transporter names are written in grey). The *P. armoraciae*-specific clade investigated in this study is highlighted with a grey background. The tissue-specific localization of three sugar porters from *C. populi* that are closely related to *Pa*GTRs is written in parentheses behind the transporter names. MT, Malpighian tubules; FB, fat body. Bootstrap support values higher than 50% are indicated on the corresponding branches. The tree was rooted with a putative vesicular neurotransmitter transporter (Transporter Classification Database ID 2.A.1.14) from *P. armoraciae* (*Pa*2537-1).

Supplementary Tables 1-5

Supplementary Table 1. Glucosinolate (GLS) concentrations in the hemolymph of seven-day old adult *P. armoraciae*.

Glucosinolate	Glucosinolate concentration (mM)						
	Replicate 1	Replicate 2	Replicate 3	Replicate 4	Replicate 5	Replicate 6	Mean ± SD
2Prop GLS	55.769	59.682	97.330	67.331	67.277	80.198	71.265 ± 15.263
3But GLS	1.081	4.937	3.670	3.791	3.725	2.373	3.263 ± 1.343
2PE GLS	0.157	0.302	0.938	0.704	0.280	0.342	0.454 ± 0.300
Benzyl GLS	0.345	0.386	0.634	0.735	0.401	0.258	0.460 ± 0.184
I3M GLS	1.948	1.811	3.118	2.626	1.289	1.702	2.082 ± 0.668
Total	59.300	67.118	105.689	75.187	72.972	84.873	77.523 ± 16.210

2Prop, 2-propenyl; 3But, 3-butenyl; 2PE, 2-phenylethyl; I3M, indol-3-ylmethyl.

Supplementary Table 2. Glucosinolate levels in *PaGTR1*-silenced and control beetles.

**Glucosinolate**	**nmol sequestered glucosinolate per adult (mean ± SD; *n* = 10)**		**Statistical method[1]**	**Statistics**	***P* value**
	dsIMPI	***dsPaGTR1***			
3MSOP GLS	1.231 ± 0.195	1.394 ± 0.282	Two-tailed Student's *t*-test	*t* = -1.507	0.149
3MTP GLS	0.188 ± 0.051	0.232 ± 0.088	Two-tailed Student's *t*-test	*t* = -1.343	0.196
4MSOB GLS	3.487 ± 0.491	4.087 ± 1.096	Two-tailed Student's *t*-test	*t* = -1.581	0.131
4MTB GLS	10.256 ± 2.100	11.200 ± 3.071	Two-tailed Student's *t*-test	*t* = -0.803	0.433
5MSOP GLS	0.191 ± 0.046	0.222 ± 0.032	Two-tailed Student's *t*-test	*t* = -1.753	0.097
7MSOH GLS	0.119 ± 0.032	0.158 ± 0.030	Two-tailed Student's *t*-test	*t* = -2.830	0.011
8MSOO GLS	1.135 ± 0.286	1.306 ± 0.441	Two-tailed Student's *t*-test	*t* = -1.028	0.317
2Prop GLS	55.841 ± 7.800	52.509 ± 3.984	Mann-Whitney *U* test	*U* = 38.000	0.385
3But GLS	0.935 ± 0.161	1.007± 0.295	Two-tailed Student's *t*-test	*t* = -0.683	0.503
I3M GLS	2.830 ± 0.467	0.889 ± 0.303	Two-tailed Student's *t*-test	*t* = 10.975	< 0.001
4OHI3M GLS	0.002 ± 0.004	0.002 ± 0.002	Mann-Whitney *U* test	*U* = 50.000	1
4MOI3M GLS	0.159 ± 0.084	0.227 ± 0.095	Two-tailed Student's *t*-test	*t* = -1.687	0.109
1MOI3M GLS	0.026 ± 0.008	0.004 ± 0.002	Mann-Whitney *U* test	*U* = 0.000	< 0.001
Total	76.399 ± 8.738	73.248 ± 4.350	Two-tailed Student's *t*-test	*t* = 1.021	0.321

[1]Analyses were performed using SigmaPlot 14.0. 3MSOP, 3-methylsulfinylpropyl; 3MTP, 3-methylthiopropyl; 4MSOB, 4-methylsulfinylbutyl; 4MTB, 4-methylthiobutyl; 5MSOP, 5-methylsulfinylpentyl; 7MSOH, 7-methylsulfinylheptyl; 8MSOO, 8-methylsulfinyloctyl; 2Prop, 2-propenyl; 3But, 3-butenyl; I3M, indol-3-ylmethyl; 4OHI3M, 4-hydroxyindol-3-ylmethyl; 4MOI3M, 4-methoxyindol-3-ylmethyl; 1MOI3M, 1-methoxyindol-3-ylmethyl.

Supplementary Table 3. Glucosinolate levels in *PaGTR5/6/7/8*-silenced and control beetles.

Glucosinolate	nmol sequestered glucosinolate per adult (mean ± SD; n = 10)		Transformation	Statistics[1]	P value
	dsIMPI	***dsPaGTR5/6/7/8***			
3MSOP GLS	1.859 ± 0.217	1.713 ± 0.251	-	t = 1.388	0.182
3MTP GLS	0.432 ± 0.098	0.318 ± 0.087	-	t = 2.739	0.013
4MSOB GLS	4.555 ± 0.802	4.582 ± 0.855	-	t = -0.073	0.943
4MTB GLS	14.793 ± 1.872	12.287 ± 2.012	-	t = 2.882	0.010
5MSOP GLS	0.295 ± 0.048	0.315 ± 0.077	Square-root	t = -0.648	0.525
7MSOH GLS	0.218 ± 0.026	0.220 ± 0.045	-	t = -0.139	0.891
8MSOO GLS	1.317 ± 0.195	1.281 ± 0.376	-	t = 0.263	0.795
2Prop GLS	54.667 ± 5.272	47.919 ± 5.412	-	t = 2.825	0.011
3But GLS	1.287 ± 0.360	1.456 ± 0.584	Log_{10}	t = -0.791	0.439
I3M GLS	4.227 ± 0.487	5.225 ± 0.907	-	t = -3.069	0.007
4MOI3M GLS	0.301 ± 0.140	0.440 ± 0.173	-	t = -1.977	0.064
1MOI3M GLS	0.070 ± 0.010	0.070 ± 0.017	-	t = -0.010	0.992
Total	84.021 ± 5.319	75.828 ± 4.321	-	t = 3.781	0.001

[1]Analyses were performed with two-tailed Student's *t*-test using SigmaPlot 14.0. 3MSOP, 3-methylsulfinylpropyl; 3MTP, 3-methylthiopropyl; 4MSOB, 4-methylsulfinylbutyl; 4MTB, 4-methylthiobutyl; 5MSOP, 5-methylsulfinylpentyl; 7MSOH, 7-methylsulfinylheptyl; 8MSOO, 8-methylsulfinyloctyl; 2Prop, 2-propenyl; 3But, 3-butenyl; I3M, indol-3-ylmethyl; 4MOI3M, 4-methoxyindol-3-ylmethyl; 1MOI3M, 1-methoxyindol-3-ylmethyl.

Supplementary Table 4. Gene expression variability across tissues among four tested reference genes.

**Target gene**	**Tissue**	**Mean Cq value of four biological replicates**	**Standard deviation of the mean Cq values**
	Foregut	21.70	
	Midgut	21.96	
eIF4A	Hindgut	21.62	0.30
	Malpighian tubules	21.39	
	Other tissues	21.17	
	Foregut	20.81	
	Midgut	20.44	
RPL13a	Hindgut	20.98	0.31
	Malpighian tubules	20.22	
	Other tissues	20.49	
	Foregut	21.42	
	Midgut	20.39	
RPL7	Hindgut	21.12	0.42
	Malpighian tubules	20.63	
	Other tissues	20.62	
	Foregut	21.37	
	Midgut	20.44	
RPS4e	Hindgut	21.39	0.46
	Malpighian tubules	20.77	
	Other tissues	20.52	

Supplementary Table 5. Multiple reaction monitoring (MRM) transitions for compounds determined by LC-MS/MS.

Compound	Q1 [m/z]	Q3 [m/z]	CE [eV]	Use
2Prop GLS	358	95.9	-60	All samples except those of the pH-dependency experiment using the *Xenopus* oocyte expression system
4MSOB GLS	435.9	95.8	-60	
4MTB GLS	419.9	95.9	-58	
2PE GLS	421.81	95.9	-50	
Benzyl GLS	408	95.9	-60	
4OHBenz GLS	424	95.9	-60	
I3M GLS	447	95.8	-50	
3But	372	95.9	-60	
Salicin	285	123	-18	
Dhurrin	310	179	-10	
Linamarin (formiate adduct)	292	45	-26	
Aucubin (formiate adduct)	391	183	-18	
Catalpol (formiate adduct)	407	199	-18	
2Prop GLS	358	97.0[Q]	22	Samples of the pH-dependency experiment using the *Xenopus* oocyte expression system
	358	75	30	
	358	259	20	
I3M GLS	447	97.0[Q]	10	
	447	259	10	
	447	205	10	

[Q]quantifier ion, additional transitions are used for identification only. 2Prop, 2-propenyl; 4MSOB, 4-methylsulfinylbutyl; 4MTB, 4-methylthiobutyl; 2PE, 2-phenylethyl; 4OHBenz, 4-hydroxybenzyl; I3M, indol-3-ylmethyl; 3But, 3-butenyl.
