# Supplementary material for: Sugar transporters enable a leaf beetle to accumulate plant defense compounds
Source: Nat Commun. 2021 May 11;12:2658. doi: 10.1038/s41467-021-22982-8 (PMC8113468; doi:10.1038/s41467-021-22982-8)
Supplement: Supplementary file 2 — Description of Additional Supplementary Files [file 41467_2021_22982_MOESM2_ESM.docx]

**Description of Additional Supplementary Files**

Supplementary Data 1

Description: Numbers of putative transporters in the *Phyllotreta armoraciae* transcriptome predicted by the Transporter Automatic Annotation Pipeline

Supplementary Data 2

Description: MFS transporters identified in the transcriptome of *Phyllotreta armoraciae* and the genomes of *Leptinotarsa decemlineata*, *Anoplophora glabripennis* and *Tribolium castaneum*

Supplementary Data 3

Description: Phylogenetic tree of MFS transporters from four beetle species

Supplementary Data 4

Description: Primers used in this study

Supplementary Data 5

Description: *In silico* off-target prediction of dsRNA targets against the local *Phyllotreta armoraciae* transcriptome database

Supplementary Data 6

Description: Statistical data

Supplementary Data 7:

Description: Predicted protein dataset of *Tribolium castaneum* assembly Tcas3.0
