# Supplementary material for: Sugar transporters enable a leaf beetle to accumulate plant defense compounds
Source: Nat Commun. 2021 May 11;12:2658. doi: 10.1038/s41467-021-22982-8 (PMC8113468; doi:10.1038/s41467-021-22982-8)
Supplement: Supplementary file 5 — Supplementary Data 3 [file 41467_2021_22982_MOESM5_ESM.pdf]

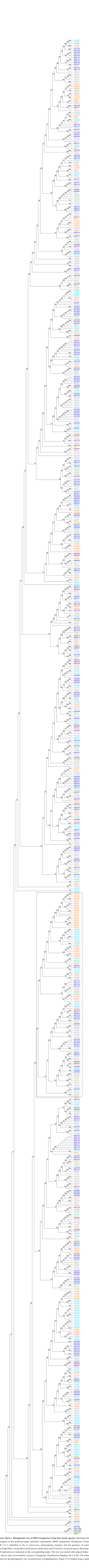

Supplementary Data 3. Phylogenetic tree of MFS transporters from four beetle species. Maximum-likelihood inferred phylogeny of the predicted major facilitator superfamily (MFS) transporters (Transporter Classification Database ID 2.A.1) identified in the *P. armoraciae* transcriptome (orange) and the genomes of *Leptinotarsa decemlineata* (light blue), *Anoplophora glabripennis* (dark blue), and *Tribolium castaneum* (grey). Bootstrap support values (1000 replicates) are indicated on the corresponding nodes. The tree was rooted with reduced folate carriers from *Homo sapiens* and *Caenorhabditis elegans* (Transporter Classification Database ID 2.A.48). The subset of the sequences used for the phylogenetic tree reconstruction in Supplementary Figure 10 is framed using a dashed line.
