# Supplementary material for: Sugar transporters enable a leaf beetle to accumulate plant defense compounds
Source: Nat Commun. 2021 May 11;12:2658. doi: 10.1038/s41467-021-22982-8 (PMC8113468; doi:10.1038/s41467-021-22982-8)
Supplement: Supplementary file 10 — Reporting Summary [file 41467_2021_22982_MOESM10_ESM.pdf]

nature research

Corresponding author(s): Franziska Beran
Last updated by author(s): Mar 27, 2021

Reporting Summary

Nature Research wishes to improve the reproducibility of the work that we publish. This form provides structure for consistency and transparency in reporting. For further information on Nature Research policies, see our Editorial Policies and the Editorial Policy Checklist.

Statistics

For all statistical analyses, confirm that the following items are present in the figure legend, table legend, main text, or Methods section.

n/a	Confirmed	
☐	☒	The exact sample size (*n*) for each experimental group/condition, given as a discrete number and unit of measurement
☐	☒	A statement on whether measurements were taken from distinct samples or whether the same sample was measured repeatedly
☐	☒	The statistical test(s) used AND whether they are one- or two-sided<br>*Only common tests should be described solely by name; describe more complex techniques in the Methods section.*
☒	☐	A description of all covariates tested
☐	☒	A description of any assumptions or corrections, such as tests of normality and adjustment for multiple comparisons
☐	☒	A full description of the statistical parameters including central tendency (e.g. means) or other basic estimates (e.g. regression coefficient) AND variation (e.g. standard deviation) or associated estimates of uncertainty (e.g. confidence intervals)
☐	☒	For null hypothesis testing, the test statistic (e.g. *F*, *t*, *r*) with confidence intervals, effect sizes, degrees of freedom and *P* value noted<br>*Give P values as exact values whenever suitable.*
☒	☐	For Bayesian analysis, information on the choice of priors and Markov chain Monte Carlo settings
☒	☐	For hierarchical and complex designs, identification of the appropriate level for tests and full reporting of outcomes
☒	☐	Estimates of effect sizes (e.g. Cohen's *d*, Pearson's *r*), indicating how they were calculated

Our web collection on statistics for biologists contains articles on many of the points above.

Software and code

Policy information about availability of computer code

Data collection	Quantitative PCR was performed on a Bio-Rad CFX Connect Real-Time System. Analyst Software 1.6 Build 3773 (AB Sciex) or Bruker MS Workstation software (Version 8. 2.1.2, Bruker) was used for data acquisition and processing of the LC-MS/MS experiments. Chemstation for LC 3D systems Rev. A.09.01 (Agilent) was used for data acquisition and processing of the HPLC-UV experiments.
Data analysis	RNASeq data was analyzed using CLC Genomics Workbench software V 10.1 (CLC Bio). Transporters were predicted by the Transporter Automatic Annotation Pipeline (TransAAP) hosted at the TransportDB 2.0 web portal (Elbourne et al. 2017). Protein sequences were aligned using the MEGA 7 (Kumar et al. 2016). The best substitution models were determined using ProtTest 3.4.2 (Darriba et al. 2011). Phylogenetic trees were constructed in IQ-TREE version 1.6.0 (Nguyen et al. 2015). The tree was visualized using FigTree 1.4.3 (http://tree.bio.ed.ac.uk/software/figtree/). The steady-state kinetic analysis was conducted in SigmaPlot 14.0 (Systat Software Inc.). Areas of leaves were measured by Adobe Photoshop CS5 (Adobe Systems Inc). Statistical analyses were conducted in R 3.5.1 (R Core Team, 2018) or in SigmaPlot 14.0 or 14.5 (Systat Software, Inc). Transmembrane domains of proteins were predicted by TMHMM 2.0 (http://www.cbs.dtu.dk/services/TMHMM/). Primers were designed using primer3 (https://primer3.ut.ee/). The local protein sequence search was conducted using BlastP (Altschul et al. 1990).

For manuscripts utilizing custom algorithms or software that are central to the research but not yet described in published literature, software must be made available to editors and reviewers. We strongly encourage code deposition in a community repository (e.g. GitHub). See the Nature Research guidelines for submitting code & software for further information.

Data

Policy information about availability of data

All manuscripts must include a data availability statement. This statement should provide the following information, where applicable:

- Accession codes, unique identifiers, or web links for publicly available datasets
- A list of figures that have associated raw data
- A description of any restrictions on data availability

Source data for main figures and supplementary figures are provided with the paper. The sequences of the genes cloned in this paper have been deposited in the GenBank database (accession nos. MN433061–MN433082). RNASeq raw data used to assemble the transcriptome have been deposited in the sequence read archive (SRA) database (Accession Number: PRJNA716471). Other data that support the findings of this study are available from the corresponding author upon request.

Field-specific reporting

Please select the one below that is the best fit for your research. If you are not sure, read the appropriate sections before making your selection.

[x] Life sciences   [ ] Behavioural & social sciences   [ ] Ecological, evolutionary & environmental sciences

For a reference copy of the document with all sections, see nature.com/documents/nr-reporting-summary-flat.pdf

Life sciences study design

All studies must disclose on these points even when the disclosure is negative.

Sample size	No statistical methods were used to predetermine sample size. Sample sizes were chosen based on previous studies and literature using similar methods.
Data exclusions	Data points were excluded when there was a technical mistake during the experiment.
Replication	We performed all experiments with at least three biological replicates. All attempts at replication were successful.
Randomization	Samples were assigned to different experimental groups randomly.
Blinding	Due to the nature of the experimental design blinding was not practical in this study.

Reporting for specific materials, systems and methods

We require information from authors about some types of materials, experimental systems and methods used in many studies. Here, indicate whether each material, system or method listed is relevant to your study. If you are not sure if a list item applies to your research, read the appropriate section before selecting a response.

Materials & experimental systems

n/a	Involved in the study	
[ ]	[x]	Antibodies
[ ]	[x]	Eukaryotic cell lines
[x]	[ ]	Palaeontology and archaeology
[ ]	[x]	Animals and other organisms
[x]	[ ]	Human research participants
[x]	[ ]	Clinical data
[x]	[ ]	Dual use research of concern

Methods

n/a	Involved in the study	
[x]	[ ]	ChIP-seq
[x]	[ ]	Flow cytometry
[x]	[ ]	MRI-based neuroimaging

Antibodies

Antibodies used	Anti-His (C-Term)-HRP Antibody was purchased from Novex, Life technologies, under LOT number 2146784, REF number 46-0707.
Validation	The antibody is commercially available and was validated by the manufacturer.

Eukaryotic cell lines

Policy information about cell lines

Cell line source(s)	The High Five insect cells were purchased from Gibco, under catalog number B85502.
Authentication	We did not perform authentication for the cell line used in this study.
Mycoplasma contamination	The cell line was not tested for Mycoplasma contamination.
Commonly misidentified lines (See ICLAC register)	The study did not involve any commonly misidentified lines.

Animals and other organisms

Policy information about studies involving animals; ARRIVE guidelines recommended for reporting animal research

Laboratory animals	We only used invertebrate in this study. Larvae and adults of Phyllotreta armoraciae (females and males) used in the experiments were maintained in the Max-Planck Institute for Chemical Ecology, Jena, Germany.
Wild animals	We did not use wild animals in this study.
Field-collected samples	We did not use field-collected samples.
Ethics oversight	Ethical approval or guidance was not required.

Note that full information on the approval of the study protocol must also be provided in the manuscript.
